# Opportunities in childhood Sjögren’s disease: results from collaborative roundtable discussions

**DOI:** 10.1093/rheumatology/keae258

**Published:** 2024-05-10

**Authors:** Erin B Treemarcki, Scott M Lieberman, Matthew L Basiaga, Janet Orrock, Cuoghi Edens, Akaluck Thatayatikom, Hemalatha Srinivasalu, Claudia Bracaglia, Yuzaburo Inoue, Marija Jelusic, Jessica L Bloom, Amanda Robinson, Jessica Nguyen, Ellen Go, Penelope Martinez, Rachel L Randell, Sharon Tiger, Grace Tiger, Jamie Diianni, Seunghee Cha, Simone Appenzeller, Nora G Singer, Sara M Stern

**Affiliations:** Department of Pediatrics, University of Utah, Salt Lake City, UT, USA; Stead Family Department of Pediatrics, University of Iowa Carver College of Medicine, IA, USA, Iowa City; Department of Pediatrics and Adolescent Medicine, Mayo Clinic, Rochester, MN, USA; Department of Pediatrics, The University of Texas at Austin Dell Medical School, Austin, TX, USA; Department of Internal Medicine and Pediatrics, The University of Chicago Medical Center, Chicago, IL, USA; Department of Pediatrics, AdventHealth for Children, Orlando, FL, USA; Pediatric Rheumatology, Children’s National Hospital; Associate Professor of Pediatrics, GW School of Medicine, Washington, DC, USA; Division of Rheumatology, Ospedale Pediatrico Bambino Gesù IRCCS, Rome, Italy; Department of General Medical Science, Graduate School of Medicine, Chiba University, Chiba, Japan; Department of Paediatrics, University of Zagreb School of Medicine, University Hospital Centre Zagreb, Zagreb, Croatia; Department of Pediatrics, University of Colorado School of Medicine, Aurora, CO, USA; Department of Pediatrics, University of Utah, Salt Lake City, UT, USA; Department of Rheumatology, Texas Children’s Hospital, Houston, TX, USA; Department of Pediatrics, Indiana University School of Medicine, IN, USA; Department of Pediatrics, Icahn School of Medicine at Mount Sinai, NY, USA; Department of Pediatrics, Duke University School of Medicine, Durham, NC, USA; Childhood Arthritis and Rheumatology Research Alliance (CARRA), Beltsville, MD, USA; Childhood Arthritis and Rheumatology Research Alliance (CARRA), Beltsville, MD, USA; Childhood Arthritis and Rheumatology Research Alliance (CARRA), Broomall, PA, USA; Seunghee Cha, Oral Maxillofacial Diagnostic Sciences, University of Florida, Gainesville, FL, USA; Department of Orthopedics, Rheumatology and Traumatology, School of Medical Science, University of Campinas, SP, Brazil; Department of Medicine and Pediatrics, MetroHealth Medical Center & Case Western Reserve University, Cleveland, OH, USA; Department of Pediatrics, University of Utah, Salt Lake City, UT, USA

## Abstract

**Figure:**
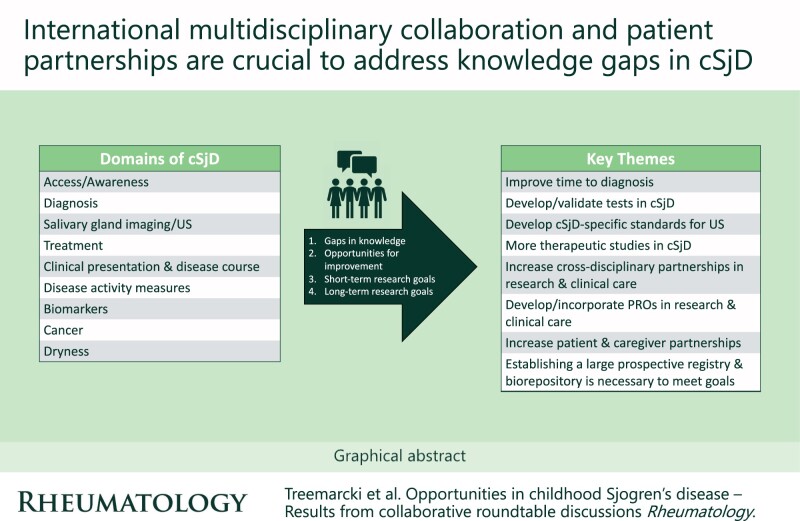

Rheumatology key messages

- International multidisciplinary collaboration and patient partnerships are essential to prioritize and address knowledge gaps in cSjD.
- This international collaborative workgroup recommends large prospective cohort studies to tackle the current most pressing knowledge gaps.

Dear Editor, In March 2023 the Childhood Sjögren’s Disease (cSjD) Workgroup met during the Childhood Arthritis and Rheumatology Research Alliance (CARRA) Annual Scientific Meeting to review the current state of cSjD and identify opportunities to prioritize this rare and understudied disease. A total of 23 stakeholders representing four continents contributed, thus representing the largest collaborative workgroup specifically dedicated to cSjD internationally. The workgroup included pediatric rheumatologists, oral health specialists, one patient and two caregivers. Here we present the findings from this working meeting.

Prior to the meeting, the following nine domains were identified by core members of the cSjD workgroup along with relevant literature: access/awareness, diagnosis, salivary gland ultrasound, treatment, clinical presentation and disease course, disease activity measures/outcome measures, biomarkers, cancer and dryness. Literature pertaining to each domain was reviewed at the meeting in order to identify: (i) gaps in knowledge; (ii) opportunities for improvement; (iii) short-term research goals; and (iv) long-term research goals. These topics were successfully identified through extensive small and large group discussions (Table 1).

**Table 1.** Key themes and research questions from nine domains of cSjD

Domain	Knowledge gaps	Opportunities for improvement	Short-term research goals	Long-term research goals
Access/Awareness	Appropriate referral to subspecialties. Transition to adult care. Timely referral to pediatric rheumatology.	Improve education among subspecialty providers & pediatric and adult rheumatologists. Increase provider awareness of cSjD.	Create educational toolkit and give educational sessions. Generate literature (case series) to support use of medications/insurance approval.	Prospective study data collection. Determine whether improved education decreases barriers to care.
Diagnosis	Heterogeneous disease. No pediatric diagnostic criteria. Diagnostic testing not validated in children.	Create a pediatric-specific classification criterion. Develop and use a multidisciplinary approach for care.	Establish pediatric-specific normative data for diagnostic testing. Increase relationship and collaboration with ophthalmologist and otolaryngologist.	Create pediatric-specific classification criteria. Develop criteria that allow for early diagnosis prior to tissue damage and dryness.
Salivary gland imaging/ultrasound	No pediatric standards or a unified approach to salivary gland ultrasound. No normative data for healthy children.	Describe a standard approach to salivary gland ultrasound. Describe normative data for healthy children. Create pediatric standards and a unified approach for cSjD.	Describe a standard approach to salivary gland ultrasound. Evaluate if OMERACT scoring system applies to children.	Describe normal salivary ultrasound findings by age and sex. Develop cSjD-specific salivary gland ultrasound disease activity and damage scoring system.
Treatment	Limited data on pharmacologic intervention and effectiveness. No FDA-approved medications. <18yo not included in adult SjD trials	Pharmacologic case-series. Replicate adult pharmacologic trials in pediatrics. Advocate for <18yo trial enrolment.	Recruit patients for prospective registry. Evaluate pharmacologic intervention used by patients in prospective registry and case series.	More pharmacologic intervention trials. Creation of consensus treatment plans. Evaluate the effect of pharmacologic intervention on fatigue, QoL, lymphoma, oral health, ocular health.
Presentation and clinical course	Lack understanding in disease progression and extraglandular manifestation.	Large prospective longitudinal study.	Recruit international participation for prospective registry.	Create validated outcome QoL, patient related outcome measures. Start to analyse data from international prospective registry.
Disease activity/outcome measures	Disease activity and outcome measures not geared to childhood disease and not validated in children.	Development of pediatric-specific disease activity, damage and patient relevant outcome measures.	Attempt to validate ESSPRI in pediatric age group. Create questionnaire to evaluate important QoL aspects in cSjD.	Develop and validate a pediatric-specific measure of disease activity, damage and outcomes. Create patient-related outcome measures based on patient & caregiver importance.
Biomarkers	Paucity of studies.	More studies focusing on biomarkers.	Sample collection (saliva, blood, and tears).	Biomarker development for diagnostics. Disease activity scale creation. Evaluate malignancy & dryness risk.
Cancer	Unknown risk of cancer in cSjD.	Multidisciplinary partnership for treatment and research collaboration.	Creating multidisciplinary partnership. Educational campaign.	Study if there is increased risk for MALT lymphoma in cSjD and estimate risk.
Dryness	Unknown risk of dryness progression in cSjD.	Multidisciplinary partnership for treatment and research collaboration.	Creating multidisciplinary partnership. Educational campaign.	Discover risk factors for dryness progression. Develop biomarkers for disease activity and risk factors for progression.

cSjD: Childhood Sjögren’s disease; FDA: Food and Drug Administration; OMERACT: Outcome Measures in Rheumatoid Arthritis Clinical Trials; QoL: quality of life; SjD: Sjögren’s disease.

Key themes and research questions emerged including but not limited to the following:

- **Diagnosis** of cSjD is challenging and often delayed. Contributing factors include the lack of pediatric-specific diagnostic criteria and differing presentations in children versus adults [1].
- **Objective tests** used to assess SjD in adults have not been validated and may be unreliable, difficult and/or painful in some children. Measures for disease monitoring and diagnosis to assess cSjD need to be feasible in a pediatric clinical care setting and better tolerated by children.
- There is currently no sensitive **test to rule out/rule in cSjD**. Many of the diagnostic tests for cSjD depend on sicca, which is often absent in cSjD. We emphasize that research that could uncover disease activity biomarkers as well as biomarkers seen early in the course of disease need to be a priority. The group encourages the study of saliva-based biomarkers as a less invasive testing method. Forming a biorepository is a necessary first step [1, 2, 4–7].
- **Salivary gland ultrasound** is a noninvasive test used in cSjD; yet there are no pediatric standards or unified approaches to salivary gland ultrasound in this disease. Research is needed to create a formal approach for salivary gland ultrasound in cSjD. Importantly, normative data on salivary gland ultrasound for healthy children by age and sex is needed prior to the development of cSjD-specific standards [1, 3, 5, 7, 8].
- Pediatric rheumatologists are using a wide variety of medications in the treatment of cSjD despite a lack of published reports on effectiveness of these medications. The group stresses the necessity for **more therapeutic studies** in cSjD. The group agreed that results from adult medication trials may not translate to children and recommend replicating these trials in the pediatrics population [1, 5].
- Understanding the **risk of MALT lymphoma** and other malignancies in cSjD is a high priority for all stakeholders, which will require a long-term follow-up and collaboration with oncologists, otolaryngologists and adult rheumatologists [1, 7].
- **Cross-disciplinary partnerships** are essential in diagnosing and managing cSjD due to the diversity of presentations and multisystem involvement. We recommend partnering with ophthalmologists, otolaryngologists, radiologists and oral medicine specialists to normalize diagnostic testing, develop diagnostic resources for providers and patients, and increase education, awareness and access. We also recommend a multidisciplinary transition of care plan for when they transfer to adult care [1–4].
- Given the chronicity of this condition, research should focus on areas that can improve the **quality of life** (QoL) of patients with cSjD. Patient reported outcomes (PROs) should, therefore, be routinely incorporated into research to understand the impact of this multisystem disease. Due to the disease variability over time, PRO measures need to have the flexibility to capture the cumulative disease burden for each patient. Measures to assess dryness, fatigue and pain need to be developmentally appropriate and directed toward patients/caregivers [1, 2, 5].
- **Patient and caregiver research partners** are essential components of any research team. In addition to helping derive clinically relevant questions, their involvement helps ensure study design does not overburden participants, identifies meaningful study outcomes, and aids in study recruitment. Patient and caregiver partners may also contribute to data analysis, dissemination of knowledge gained and future research directions.
- **Proposed long-term goals** include conducting research on cSjD outcomes, developing a pediatric-specific diagnostic criteria and improving diagnosis and treatment before damage develops. The group agrees that a large prospective registry is necessary to achieve these goals and international collaboration is vital [1, 2, 5].

Two unifying themes developed in identifying our research path forward in cSjD. First, research projects should be relevant to pediatric patients and improve their quality of life directly or indirectly. Second, many of our questions will take time to answer, and we collectively agree that a large prospective cohort study is needed to increase our understanding of many key aspects of cSjD. The group highlights the importance of global international collaboration to increase the power of our studies to provide quality data to children with cSjD.

## Data Availability

No new data were generated or analysed in support of this research.
